# A Word as to Legislation

**Published:** 1897-04

**Authors:** 


### EDITORIAL.

### A WORD AS TO LEGISLATION.

WE hear and receive many warm commendations on the progress of veterinary legislation in Pennsylvania, and we fear that there is a growing impression that we are specially favored in the Keystone State. We do not want to discourage any of the workers in sister States, but we feel it incumbent to say that we have learned well the adage that "God helps those who help themselves." Every concession in Pennsylvania has been won after a long, earnest, and persistent battle. The veterinary profession of the Keystone State has a number of tireless leaders, full of energy and determination, aided and seconded by a united profession, ever ready to give personal and financial assistance. The battles have been fought to win, and well may the profession of this State be proud of the enactments engrafted upon the State statutes. The annual meeting of the Association just closed was one of the most fruitful ever held, and the members determined there to again urge greater assistance to the State Live-stock Sanitary Board and to join the State Grange and dairymen in securing the passage of a law requiring that animals entering our State to replenish our dairies shall have been tested or shall be submitted to a test. They voted and paid over to the State Board of Veterinary Medical Examiners one hundred and fifty dollars to be used in pressing prosecutions of violations of the State Acts relative to the practice of veterinary science. It is this plan, this persistent and ever-at-it method, that counts; and as it wins in Pennsylvania, it will win in any other State, and we most earnestly commend the profession of other States to get together, work in unison, determine just what you want and need, and go in and win.

